# Tocilizumab-Induced Acute Liver Injury in Adult Onset Still's Disease

**DOI:** 10.1155/2013/964828

**Published:** 2013-07-15

**Authors:** Michael Drepper, Laura Rubbia-Brandt, Laurent Spahr

**Affiliations:** ^1^Division of Gastroenterology and Hepatology, Department of Internal Medecine Specialties, Geneva University Hospital, Rue Gabrielle Perret Gentil 4, 1211 Geneva 14, Switzerland; ^2^Division of Clinical Pathology, Geneva University Hospital and Geneva Faculty of Medicine, Rue Gabrielle Perret Gentil 4, 1211 Geneva 14, Switzerland

## Abstract

*Background*. Tocilizumab, a monoclonal humanized anti-IL-6 receptor antibody, is used in treatment of refractory adult onset Still's disease (AOSD). Mild to moderate liver enzyme elevation is a well-known side effect, but severe liver injury has only been reported in 3 cases in the literature. *Case*. A young female suffering from corticoid and methotrexate refractory AOSD was treated by tocilizumab. After 19 months of consecutive treatment, she developed acute severe liver injury. Liver biopsy showed extensive hepatocellular necrosis with ballooned hepatocytes, highly suggestive of drug-induced liver injury. No other relevant drug exposure beside tocilizumab was recorded. She recovered totally after treatment discontinuation and an initial 3-day course of intravenous N-acetylcysteine with normalization of liver function tests after 6 weeks. *Conclusion*. Acute severe hepatitis can be associated with tocilizumab as documented in this case. Careful monitoring of liver function tests is warranted during tocilizumab treatment.

## 1. Introduction

Adult onset Still's disease (AOSD) is a systemic inflammatory disease clinically characterized by remittent fever, polyarthritis, stain rash, and lymphadenopathy in association with deregulated proinflammatory cytokines (interleukin-1, -6 and -18, tumor necrosis factor *α*, and interferon *γ*) production [1, 2]. Of interest, a correlation of IL-6 serum levels with AOSD's activity has been described [1, 3].

Tocilizumab is a neutralizing humanized monoclonal antibody against the human interleukin-6 (IL-6) receptor that is able to block soluble and membrane-bound receptors [4]. It is licensed over the world as second-line treatment for rheumatoid arthritis (RA) and in Japan and Europe for juvenile idiopathic arthritis [5, 6]. Disease improvement or remission has been reported following tocilizumab treatment in refractory AOSD [2, 7].

## 2. Case Report

An 18-year-old female from Angola was diagnosed with AOSD in September 2010 following recurrent fever, polyarthritis, abdominal lymphadenopathy, liver and spleen enlargement, and hyperferritinemia. She was initially treated with oral corticoids with early methotrexate adjunction due to incomplete control of symptoms. Despite this treatment, corticoids could not be tapered under the dose of 15 mg/day, motivating introduction of tocilizumab (8 mg/kg) in June 2011, permitting interruption of corticoids and methotrexate in December 2011. In 2012, she had no symptoms and liver function tests were normal under tocilizumab once every 3 weeks.

**Figure 1:**
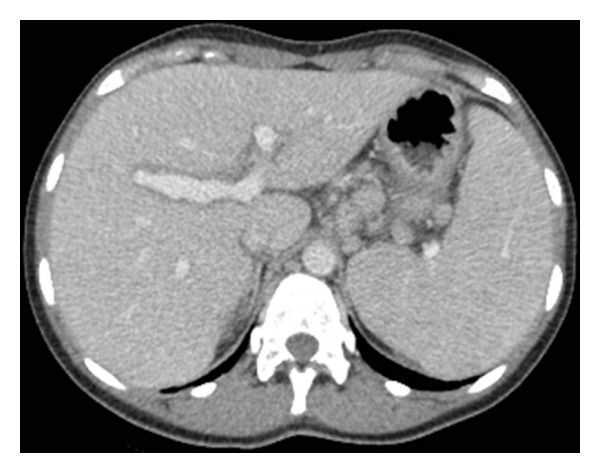
Abdominal CT showing mild liver and spleen enlargement.

**Figure 2:**
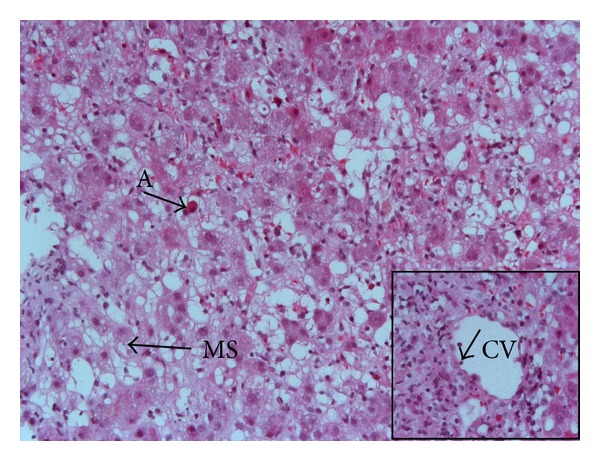
Histological view (haematoxylin-eosin stain, original magnification ×200) demonstrating extensive areas of hepatocytes with necrosis, ballooning degeneration, macro- and microvesicular steatosis (MS), and acidophil bodies (A). Insert: centrilobular vein (CV) showing endotheliitis (arrow) (original magnification ×400).

**Figure 3:**
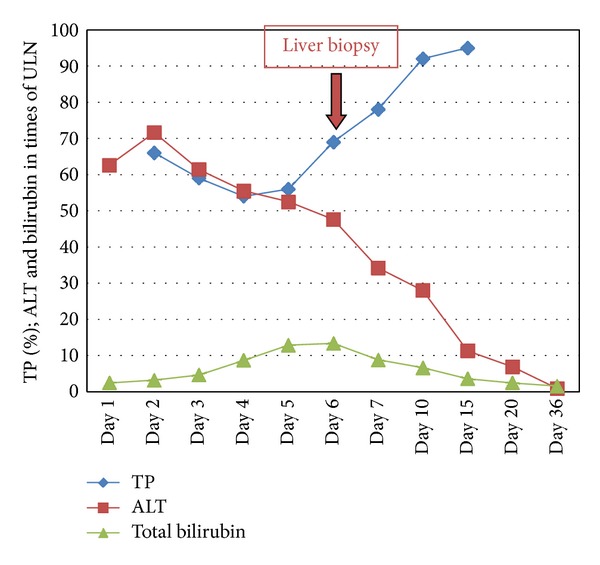
Liver function test evolution: day 1: January 24, 2013 and ULN: upper limit of normal.

On January 21, 2013, she complained of nausea, abdominal discomfort, and pruritus that persisted for two days in spite of a daily dose of 1 gram of paracetamol. On January 24, biological tests showed the following: white blood cells: 3500/*μ*L (*N*  4000–11000), platelets: 52000/*μ*L (*N*  150000–350000), aspartate aminotransferase (AST): 2622 U/L (*N*  11–42), alanine aminotransferase (ALT): 2628 U/L (*N*  9–42), alkaline phosphatase (ALP): 110 U/L (*N*  30–125), gamma glutamyltranspeptidase (GGT): 272 U/L (*N*  9–35), and total bilirubin: 61 *μ*mol/L (*N*  7–25). Prothrombin time was 66% (*N* > 70) with an International Normalized Ratio of 1.21, but dosage of factor *V* activity was normal as well as kidney function. Blood paracetamol concentration was not measured at admission due to minor exposure. At hospital admission, she was alert with a slightly tender liver and no signs of chronic liver disease. Abdominal imaging was normal except for mild liver and spleen enlargement (Figure 1). Viral hepatitis (A, B, C, and E) as well as HIV, CMV, EBV, and herpes simplex virus tested negative. Autoimmune testing (total IgG, antinuclear, antiactin, and anti-LKM1 antibodies) was negative and ceruloplasmin was normal. Transaminase levels remained elevated and bilirubin levels increased to 335 *μ*mol/L. On January 28 a transjugular liver biopsy was performed with a portosushepatic venous pressure gradient of 8 mmHg. Histology showed extensive centrilobular hepatocyte necrosis with collapse. Hepatocytes were ballooned with clear cytoplasm. Mild inflammatory periportal cell infiltrations as well as rare areas of micro- and macrovesicular steatosis were also observed (Figure 2). These histological alterations were consistent with acute drug-induced liver injury (DILI). She received a 3-day course of intravenous *N*-acetylcysteine according to the recent literature reporting transplant-free survival in acute severe nonacetaminophen liver injury [8, 9], and liver function tests were monitored until normalization 6 weeks later (Figure 3).

Causality assessment by the Naranjo score and the Liverpool Adverse Drug Reaction Causality Assessment Tool [10] both indicated tocilizumab as a probable cause of our patient's acute liver failure. The patient and her rheumatologist were instructed not to be reexposed to tocilizumab.

## 3. Discussion 

The liver has an important ability to regenerate after injury or resection with IL-6 playing a key role in its regulation. Secreted by Kupffer cells, this cytokine primes hepatocytes for mitosis, which are then stimulated by mitogenic factors like hepatic growth factor (HGF) until achieving the original liver mass again. Accordingly, IL-6 deficient mice were more vulnerable to carbon tetrachloride (CCl_4_), a molecule used to simulate drug-induced liver toxicity, with a protective effect of IL-6 injection [4, 11].

Severe liver injury is a rare complication of tocilizumab therapy. Published trials on tocilizumab in rheumatoid arthritis (RA) reported mild to moderate AST and ALT elevations in a maximum of 40 and 2.7%, respectively [12, 13], with only 3 cases of severe liver injury [14–16]. We report here a detailed case of acute DILI caused by tocilizumab with a complete resolution of liver function tests. Our observation differs from the other three cases of severe tocilizumab-induced liver injury already published. 

Mahamid et al. and Hiura et al. reported each a case with rather sub acute evolution over months leading in the first case to histological focal pericentral vein hepatocellular necrosis and steatosis [14]. In the second one, of note a lethal case, marked liver atrophy and ascites were observed with no focal necrosis but mild inflammatory cell infiltration and little periportal fibrosis at histology [15]. Interestingly, liver enzymes were normal or only slightly elevated, and supplementary immunohistochemical analysis in the second case revealed a high amount of cleaved caspase 3 antigen positive hepatocytes, suggesting rather apoptosis than necrosis at the origin of liver injury [15]. The authors hypothesized that the poor outcome could be due to IL-6 inhibition, a cytokine involved with liver regeneration [4].

On the contrary, Alfreijat et al. described one case of severe acute hepatitis similar to ours, with important liver enzyme elevation (AST 1455 U/L and ALT 2296 U/L) and hyperbilirubinemia (178.5 *μ*mol/L). Liver biopsy showed focal hepatocellular necrosis with ballooning degeneration of hepatocytes and focal bile stasis. Biological tests returned to normal after tocilizumab discontinuation and 10 weeks under steroids [16]. However, concomitant use of potentive hepatotoxic drugs rendered the causal relationship between tocilizumab and DILI more difficult in this case. Rather quick recovery in both cases favors acute tocilizumab toxicity rather than IL-6-blockage-induced impaired liver regeneration at the origin of the liver damage.

Our patient developed liver injury 19 months after initial tocilizumab exposure, a longer period compared to 1 and 3 months in previously reported cases. Nevertheless, the period from drug intake to DILI manifestation can vary considerably and is only one item among other criteria in the causality assessment scales [17, 18]. Moreover, no other relevant drug exposure has been identified, and liver biopsy showed clear signs of drug-induced liver injury.

Finally, liver enzyme elevation or liver damage may be associated with AOSD itself [19]. In our case, AOSD-related liver damage seems very unlikely as the patient did not present any clinical sign of disease activity, and biological inflammation markers were low at admission.

Acute and severe hepatitis may complicate the course of tocilizumab treatment, although this is a rare event. Pathogenic mechanisms are incompletely elucidated, but interference of tocilizumab with IL-6-mediated liver regeneration may participate. Careful monitoring of liver function tests should be applied to patients receiving tocilizumab.
